# Evaluating the optimal height for hamstring activity in the maximum-speed single-leg bridge test

**DOI:** 10.1055/a-2537-6350

**Published:** 2025-03-19

**Authors:** Yuto Sano, Masashi Kawabata, Yuki Sumiya, Yuto Watanabe, Yuto Uchida, Tomoaki Inada, Masaki Murase, Tomonori Kenmoku, Hiroyuki Watanabe, Naonobu Takahira

**Affiliations:** 1 Physical Therapy for Sports and Musculoskeletal System, Kitasato University Graduate School of Medical Sciences, Sagamihara, Japan; 2 Department of Rehabilitation, Yokohama Sports Medical Center, Yokohama, Japan; 3 89285 Department of Sports Medicine, Kitasato University Graduate School of Medical Sciences, Sagamihara, Japan; 4 38088 Department of Orthopaedic Surgery, Kitasato University School of Medicine, Sagamihara, Japan

**Keywords:** electromyography, hamstring strain injury, prevention, rehabilitation, return to sports

## Abstract

Hamstring strain injuries often occur during high-speed movements; yet, no functional test reliably induces rapid hamstring contractions. This study aimed to determine the optimal platform height in the maximum-speed single-leg bridge test to maximize hamstring activation. This cross-sectional study included 26 healthy male recreational athletes. Participants performed the maximum-speed single-leg bridge test using 20, 40, and 60 cm platforms at a maximal speed. The conventional single-leg bridge test was performed using a 60 cm platform at any speed. Measurements included buttock-raising speed; muscle activity of the semitendinosus, biceps femoris, and gluteus maximus using surface electromyography; and heel-bearing force. The maximum-speed single-leg bridge test showed significantly faster buttock-raising speeds (0.7–1.0 m/s) than the single-leg bridge test (0.5 m/s; *p* <0.01). Semitendinosus and biceps femoris muscle activities were significantly higher during the maximum-speed single-leg bridge test using 60 and 40 cm platforms (>90% maximal voluntary isometric contraction) than during the single-leg bridge test and the maximum-speed single-leg bridge test using a 20 cm platform ( *p* <0.01). Gluteus maximus muscle activity during the maximum-speed single-leg bridge test was approximately double than that during the single-leg bridge test ( *p* <0.01). The heel-bearing force was significantly higher during the maximum-speed single-leg bridge test than during the single-leg bridge test, and the maximum-speed single-leg bridge test using the 40 cm platform showed the highest force ( *p* <0.01). The maximum-speed single-leg bridge test using 40 and 60 platforms required higher hamstring activity, with faster buttock-raising speeds and greater heel-bearing force than the single-leg bridge test and the maximum-speed single-leg bridge test using the 20 platform.

## Introduction

Hamstring strain injuries (HSIs) are common in sprint-based sports, accounting for 24% of all traumatic injuries in soccer and 22% in rugby 1 2 . Recovery often requires a month or more, with severe cases taking over 6 months 3 . Additionally, the recurrence rate is over 30% 4 , posing career risks to athletes.

The HSIs typically occurs at a knee flexion angle of 5°−30° and a hip flexion angle of 40°−60° during the late swing phase or early stance phase of sprinting 5 6 7 . As sprint speed increases, the hip and knee joints reach angular velocities exceeding 650°/s for hip flexion/extension and 1000°/s for knee flexion/extension 8 , increasing strain on muscle fibers and the injury risk 9 10 . Although eccentric contraction during these phases is thought to induce HSIs, there is no consensus on the contractile elements involved. Some animal studies and computational models suggest that, during the late swing phase, the contractile elements maintain their length isometrically, while the tendons elongate, and just before ground contact, they transition to concentric contraction 11 .

The single-leg bridge test (SLBT) is commonly used to assess hamstring endurance by mimicking joint angles and biarticular movements associated with the mechanism of HSIs 12 . The SLBT provides a comprehensive assessment of the hamstring as a biarticular muscle, spanning both the hip and knee joints, which is essential for dynamic activities like sprinting. However, it remains unclear whether the 60 cm platform height maximizes hamstring activity, given that hip angles during sprinting range from 40° to 60° 6 . Moreover, since participants can select their own buttock-raising speed in the SLBT, hamstring loading may vary, potentially causing variability in test results 13 .

To address these limitations, we developed a maximum-speed single-leg bridge test (MS-SLBT), which requires participants to perform the SLBT at a maximum speed. We hypothesized that the MS-SLBT induces higher muscle activity compared to the conventional SLBT without a specified speed, potentially enhancing both test quality and reproducibility. We also hypothesized that performing the MS-SLBT on both the 40 cm and 60 cm platforms would maximize hamstring muscle activation as they approximate the optimal hip flexion angle of 45° 14 . This study aimed to determine the optimal platform height in the MS-SLBT to maximize hamstring activation.

## Materials and Methods

### Study design

This cross-sectional study was conducted in a university laboratory to compare four test conditions. The independent variables were the four test conditions: the conventional SLBT using a 60 cm platform height and the MS-SLBT using three different platform heights – 20 cm (MS20), 40 cm (MS40), and 60 cm (MS60). The recruitment period ranged from February 27 to May 30, 2024, with data collection from March 18 to June 7, 2024. All participants were informed of the benefits and risks of the study, and they provided written informed consent prior to participation. This study was approved by the relevant ethics committee (study number: 2023-039).

### Participants

Twenty-six healthy recreational male athletes were included in this study (age: 21.0±1.9 y; height: 174.1±6.2 cm; weight: 69.4±11.6 kg). Eligible participants had to be at least 18 years of age and practice resistance training and sprinting regularly (>3 h/wk). Participants were free from soft tissue and orthopedic injuries to the trunk, hips, and lower limbs at the time of testing; had no history of HSIs in the previous 18 months; and had never experienced an anterior cruciate ligament injury.

### Procedure

Data collection sessions started with a standardized warm-up, which included 2 minutes of jogging at approximately 40–50% of maximal pace, self-determined by each participant 15 , followed by 3 minutes of dynamic stretching, consisting of repeated hip flexion and extension movements, as well as knee flexion and extension movements, performed in a standing position. Measurements were taken on both sides, with the order of the left and right measurements randomized using the envelope method. The SLBT was conducted first using a 60 cm platform as this height is commonly used in the conventional SLBT and served as the control condition. The MS-SLBT was then performed on three different platform heights (MS20, MS40, and MS60) in a randomized order. The SLBT was conducted first as a baseline measurement to prevent the MS-SLBT from influencing the natural operating speed of the SLBT. For all conditions, two practice sessions preceded each measurement to confirm the movement, followed by five main test measurements. For the SLBT, the participant placed his heel on a platform at 20° of knee flexion and followed the instruction to “push down through the heel to lift their buttock off the ground as high as possible at a self-selected lifting speed.” For the MS-SLBT, the participant was instructed to “push down through the heel to lift their buttock off the ground as fast and high as possible.” Additionally, no instructions were provided on how to lower the buttocks back to the ground. The participants were instructed to keep their feet as neutral as possible to avoid changes in muscle recruitment due to tibial rotation 16 . Each trial was performed alternately on the left and right sides, with at least a 5-minute rest period between trials. The 5-minute rest period was implemented to minimize fatigue and ensure consistent performance across trials 17 . Participants were instructed to remain seated during the rest period without performing additional stretching or warm-up exercises.

### Measurements

#### Buttock-raising speed

**Fig. 1:**
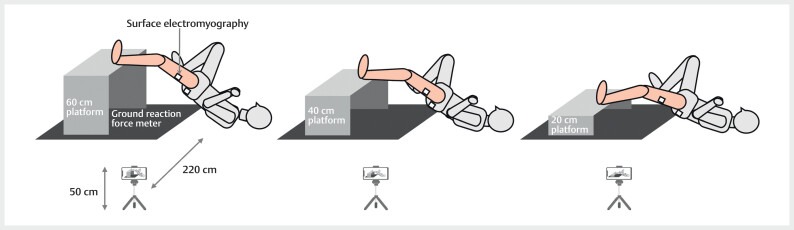
Setup of the MS-SLBT. MS-SLBT, maximum-speed single-leg bridge test.

During the SLBT and MS-SLBT, videos were recorded from the sagittal plane using an iPhone (12 Pro) positioned 220 cm from the participant and 50 cm above the ground ( Fig. 1 ). The camera was aligned parallel to the participant and recorded at 30 fps in the normal video mode.

**Fig. 2:**
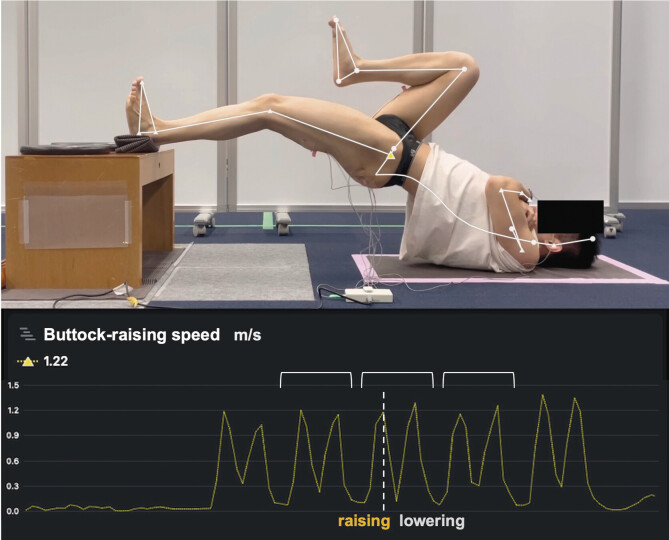
Evaluating the buttock-raising speed using an AI-based markerless motion capture analysis app for the videos taken. The lower graph represents the buttock-raising speed measured in meters per second (m/s) using SPLYZA MOTION over five consecutive trials. Each trial exhibits a biphasic pattern with one peak representing the raising phase and another peak representing the lowering phase. The segments within the brackets indicate the second to fourth buttock-raising trials. The average values of these three trials were used for analysis. AI, artificial intelligence.

The buttock-raising speed was measured using SPLYZA MOTION (SPLYZA Inc, Japan), an AI-based markerless motion capture analysis application that calculates the coordinates and speed of each body part from video images ( Fig. 2 ). Data were exported in the CSV format and analyzed using Microsoft Excel 365. The coefficient of variation for the buttock-raising speed was calculated from the middle three trials for each individual. The results were 12, 12, 11, and 15% for SLBT, MS60, MS40, and MS20, respectively.

### Electromyography measurements

Surface electromyography (sEMG) (Biometrics Ltd, UK) was used to record sEMG activity from the semitendinosus (ST), biceps femoris (BF), and gluteus maximus (GM). The participants’ skin was shaved, lightly abraded with an abrasive paste, and cleaned with a cotton alcohol wipe before the electrodes were placed. The electrodes (Biometrics Ltd, UK) (37 × 20 × 6 mm) were placed parallel to the direction of the muscle fibers at positions confirmed on palpation of the muscle belly, according to the Surface Electromyography for the Noninvasive Assessment of Muscles guidelines, and secured with tape to minimize motion artifacts 18 19 . Normalization was performed using maximal voluntary isometric contraction (MVIC). The procedure followed Noraxon’s guidelines 20 , with the participant in a prone position on the examination table. After checking the quality of the sEMG signals for each channel, participants performed two warm-up contractions followed by three 3- to 4-second MVIC trials for the hamstrings and GM, with 30 seconds of rest between trials. For the hamstrings, the participants fixed their knee to 30°, fixed the distal leg with a band, and performed the MVIC in the direction of the knee flexion. To measure the maximal muscle activity in BF and ST, the maximal power output was measured in the intermediate position in the first session, tibial external rotation in the second session, and tibial internal rotation in the third session 16 . For GM, the distal thigh was resisted with a band at 90° knee flexion and neutral hip flexion, and MVIC was performed in the direction of the hip extension.

### Heel-bearing force

The heel-bearing force was measured using a ground reaction force meter (Advanced Mechanical Technology, Inc., USA) to determine the vertical components of the force. It was set to zero when the heel was at rest on the platform.

### Data analysis

All sEMG and ground reaction force data were sampled at 1 kHz using a 16-bit PowerLab 8/30 26T AD unit (AD Instruments, New South Wales, Australia) and analyzed using LabChart 7.3.7 (AD). Raw sEMG data were filtered using a bandpass filter at 20–500 Hz, and the root mean square was calculated in a 50 ms window. The peak value was extracted from the smoothed sEMG signal of each muscle MVIC trial, and this value was defined as 100%MVIC. The buttock-raising speed, heel-bearing force, and muscle activity during the SLBT and MS-SLBT were calculated by considering the maximum values during each trial’s buttock-raising phase. Muscle activity was determined by normalizing the filtered sEMG signal to the values obtained during the MVIC and the normalized sEMG (nEMG) values averaged over the middle three trials (trials 2, 3, and 4), excluding trials 1 and 5.

### Statistical analysis

A sample size calculation using G*Power software (v3.1.9.2, University of Kiel, Germany) determined that 24 participants were needed to achieve 80% power to detect a medium effect size ( *f* = 0.25) at a 0.05 significance level, based on differences in nEMG amplitudes of BF and ST at varying speeds 21 .

Data were analyzed using JMP Pro 17 software (SAS Institute Inc.) and presented as mean, standard error of the mean, and 95% confidence interval. Before analyses, normality was determined using the Shapiro–Wilk test. A one-way repeated measures analysis of variance examined differences in buttock-raising speed, heel-bearing force, and nEMG among the three muscles between the four conditions. Statistical significance was set at *p* <0.05. When a significant main effect was detected for conditions, post hoc *t* -tests with Bonferroni correction were used to determine the source. An adjusted *p* -value of *p* <0.008 was calculated by dividing the significance level of 0.05 by the number of comparisons (six comparisons across four conditions).

## Results

Significant differences in buttock-raising speed were observed between the four conditions in the dominant leg (DL; *p* <0.001) and non-dominant leg (NDL; *p* <0.001; Table 1 ). Multiple comparisons showed significantly faster execution in the MS-SLBT than in the SLBT under all conditions ( *p* <0.001). Significantly faster executions were also observed with MS60 and MS40 than with MS20 ( *p* <0.001).

**Table:** **Table 1** Comparison of buttock-raising speeds (m/s) under different conditions.

Side	(a) SLBT	(b) MS60	(c) MS40	(d) MS20	*F* -value	*p* -value
DL (m/s)	0.45±0.02 (0.40–0.50)	0.93±0.04 ^a,d^ (0.84–1.02)	0.98±0.04 ^a,d^ (0.91–1.05)	0.74±0.04 ^a^ (0.67–0.82)	81.1	<0.001
NDL (m/s)	0.46±0.02 (0.42–0.50)	1.00±0.03 ^a,d^ (0.94–1.06)	0.97±0.03 ^a,d^ (0.91–1.03)	0.74±0.03 ^a^ (0.67–0.81)	114.1	<0.001

Abbreviations: DL, dominant leg; MS20, maximum-speed single-leg bridge test using a 20 cm platform; MS40, maximum-speed single-leg bridge test using a 40 cm platform; MS60, maximum-speed single-leg bridge test using a 60 cm platform; NDL, non-dominant leg; SLBT, single-leg bridge test.

Note: Mean and standard error of the mean (95% confidence interval).

^a–d^ Significantly higher than each condition, according to the Bonferroni test ( *p* <0.008).

“ ^a–d^ ” indicates the SLBT, MS60, MS40, and MS20, respectively.

Significant differences in the ST, BF, and GM nEMG were found between the four conditions in the DL and NDL: ST ( *p* <0.001), BF (DL: *p* = 0.006, NDL: *p* = 0.002), and GM ( *p* <0.001; Table 2 ). For ST, MS60 and MS40 had significantly higher nEMG than the SLBT and MS20 in the DL ( *p* <0.001). Additionally, all conditions during the MS-SLBT had significantly higher nEMG than those of the SLBT in the NDL (MS60 and MS40: *p* <0.001; MS20: *p* <0.005). For BF, MS60 and MS40 had significantly higher nEMG than the SLBT in the DL and NDL (DL: *p* <0.001; NDL: *p* = 0.005). For GM, MS60 and MS40 had significantly higher nEMG than SLBT in the DL and NDL ( *p* <0.001). MS40 had significantly higher nEMG than MS60 in the NDL ( *p* <0.001).

**Table:** **Table 2** Comparison of ST, BF, and GM nEMG (%MVIC) under different conditions.

Muscle	Side	(a) SLBT	(b) MS60	(c) MS40	(d) MS20	*F* -value	*p* -value
ST	DL (%MVIC)	74.4±4.8 (64.5–84.4)	95.1±5.5 ^a,d^ (83.8–106.3)	93.1±5.6 ^a,d^ (81.6–104.5)	81.3±4.5 (72.0–90.7)	14.9	< 0.001
	NDL (%MVIC)	76.1±4.5 (66.8–85.5)	92.5±6.2 ^a^ (79.8–105.2)	89.8±6.2 ^a^ (76.9–102.6)	85.8±5.5 ^a^ (74.4–97.2)	9.7	< 0.001
BF	DL (%MVIC)	102.2±6.6 (88.7–115.8)	114.8±6.3 ^a^ (101.8–127.7)	114.6±7.9 ^a^ (98.5–130.8)	106.1±7.1 (91.5–120.7)	4.5	0.006
	NDL (%MVIC)	92.3±5.5 (81.1–103.6)	107.5±6.5 ^a^ (94.0–120.9)	103.4±6.7 ^a^ (89.6–117.2)	100.3±5.5 (88.9–111.7)	5.4	0.002
GM	DL (%MVIC)	17.0±3.8 (9.3–24.8)	30.8±4.7 ^a^ (21.0–40.5)	39.6±4.4 ^a^ (30.6–48.6)	37.7±2.8 ^a^ (31.9–43.5)	18.1	< 0.001
	NDL (%MVIC)	14.9±2.1 (10.5–19.3)	28.7±3.0 ^a^ (22.4–35.0)	41.3±4.7 ^a,b^ (32.1–50.5)	37.0±3.8 ^a^ (29.1–44.9)	24.5	< 0.001

Abbreviations: BF, biceps femoris; DL, dominant leg; GM, gluteus maximus; HS20, maximum-speed single-leg bridge test using a 20 cm platform; MS40, maximum-speed single-leg bridge test using a 40 cm platform; MS60, maximum-speed single-leg bridge test using a 60 cm platform; MVIC, maximal voluntary isometric contraction; NDL, non-dominant leg; nEMG, normalized surface electromyography; SLBT, single-leg bridge test; ST, semitendinosus.

Note: Mean and standard error of the mean (95% confidence interval).

( ^a–d^ ) Significantly higher than each condition, according to the Bonferroni test ( *p* <0.008).

“ ^a–d^ ” indicates SLBT, MS60, MS40, and MS20, respectively.

Significant differences in the heel-bearing force were found between the four conditions in the DL and NDL ( *p* <0.001; Table 3 ). Multiple comparisons showed that the MS-SLBT had a significantly higher heel-bearing force than the SLBT under all conditions in the DL and NDL ( *p* <0.001), and MS40 had a significantly higher heel-bearing force than MS60 in the NDL ( *p* = 0.005).

**Table:** **Table 3** Comparison of heel-bearing force (N) under different conditions.

Side	(a) SLBT	(b) MS60	(c) MS40	(d) MS20	*F* -value	*p* -value
DL (N)	136.3±6.0 (124.0–148.7)	165.0±7.0 ^a^ (150.7–179.4)	172.7±5.1 ^a^ (162.2–183.1)	172.7±5.7 ^a^ (161.0–184.3)	29.1	< 0.001
NDL (N)	131.7±5.9 (119.4–143.9)	160.0±5.5 ^a^ (148.7–171.3)	174.6±4.9 ^a,b^ (164.5–184.7)	172.7±4.4 ^a^ (163.6–181.7)	31.1	< 0.001

Abbreviations: DL, dominant leg; MS20, maximum-speed single-leg bridge test using a 20 cm platform; MS40, maximum-speed single-leg bridge test using a 40 cm platform; MS60, maximum-speed single-leg bridge test using a 60 cm platform; NDL, non-dominant leg; SLBT, single-leg bridge test.

Note: Mean and standard error of the mean (95% confidence interval).

( ^a–d^ ) Significantly higher than each condition, according to the Bonferroni test ( *p* <0.008).

“ ^a–d^ ” indicates SLBT, MS60, MS40, and MS20, respectively.

## Discussion

This study confirmed that the buttock-raising speed in the MS-SLBT was 1.6–2.2 times faster than that in the SLBT, reaching a maximum of 1.00 m/s with MS40 and MS60. We found that the MS-SLBT requires higher hamstring and GM nEMG and a stronger heel-bearing force than the SLBT.

### Muscle activity

The MS-SLBT showed higher BF and ST nEMG than the SLBT, particularly with MS40 and MS60, with nEMG exceeding 90%MVIC. Although the SLBT reportedly has the highest nEMG (99.3%MVIC) during concentric exercises 22 , we found that the nEMG with MS40 and MS60 was even higher.

Studies have consistently shown that BF is preferentially selected during hip extension, whereas ST is preferentially selected during knee flexion 23 . The Nordic hamstring exercise, focusing on eccentric knee joint movements, results in ST muscle activity exceeding 100%MVIC, whereas the BF averages around 76.5%MVIC 22 24 . Furthermore, the 45° hip extension, which utilizes eccentric movements of the hip joint, has a BF/ST ratio that is higher than that of the Nordic hamstring exercise, but the ST muscle activity falls below 40%MVIC 22 . However, muscle activity during maximal sprinting is higher in BF during the early stance phase and in the medial hamstrings during the late swing phase 5 , with BF at 81%MVIC and ST at 108%MVIC 25 , eliciting high muscle activity in both muscles. Therefore, the MS-SLBT was a biarticular movement that recruited high muscle activity in both BF and ST, comparable with that of sprinting.

GM nEMG in the MS-SLBT was low at 30−40%MVIC but approximately twice that of the SLBT. Previous studies have reported that a GM muscle activity level of 108%MVIC is required in the late swing phase during high-speed sprinting, and athletes with HSIs show significantly lower GM muscle activity during the late swing phase and ground contact phases 26 27 . The GM is crucial for hip extension, compensating for hamstring functions 28 29 . Therefore, additional interventions targeting the GM may be necessary.

### Heel-bearing force

As the buttock-raising speed increased, the heel-bearing force in the MS-SLBT was 1.2–1.3 times greater than that in the SLBT, with MS40 showing the highest heel-bearing force in the NDL. Despite its accuracy and reproducibility, isokinetic muscle strength testing can only evaluate single knee joint movements in sitting or prone positions, and peak torque decreases significantly with increasing contraction velocity 30 . This makes it difficult to evaluate fast movements and force exertion simultaneously. Thus, the MS-SLBT could provide an additional assessment method to complement conventional functional evaluations. MS40 demonstrated a greater heel-bearing force than MS60. The optimal hip flexion angle for force exertion in BF and ST is approximately 45° 31 . This may be because the hip flexion angle in MS60 was too large to achieve maximum heel-bearing force exertion. Therefore, MS40 is the most optimal height to evaluate in positions similar to the mechanism of HSIs.

### Clinical significance

The MS-SLBT can be performed anywhere by one person with only a platform, making it a simple task. Among the tested conditions, the 40 cm platform demonstrated strong performance in terms of hamstring muscle activity and force generation. Its height also aligns with chairs and training benches commonly found in clinical and training environments, enhancing its versatility and practicality across various settings. While the 60 cm platform also showed high levels of muscle activation, the 40 cm platform offers an additional ease of implementation and accessibility, particularly in compact spaces or clinical setups. Additionally, the MS-SLBT may also be used as an exercise. Its speed of 0.97−0.98 m/s aligns with the optimal power range (0.9−1.1 m/s) found in velocity-based training for jump squats 32 33 . Thus, the MS-SLBT using a 40 cm platform effectively combines assessment and exercise for hamstring performance in clinical settings. While the 40 cm platform has demonstrated clinical utility in hamstring activation and force output, this study does not establish a direct relationship with the prevention or rehabilitation of HSIs. Future research is needed to explore this potential and validate its relevance. Moreover, the MS-SLBT could benefit not only athletes but also evaluators, such as physicians and therapists by bridging the gap in movement intensity between clinical and sports settings. This test shows promise as a tool for monitoring and supporting athletes during their return-to-sport process.

### Limitations and prospects

This study had some limitations. First, all participants were young adult males from the college sports club. Since the incidence of HSIs is influenced by age, sex and competition levels, it is uncertain whether similar responses would be observed in female athletes, more competitive athletes, or other age groups. Second, when using sEMG, there is always the possibility of crosstalk between adjacent muscles 34 . Finally, as this was a cross-sectional study, the relationship between the MS-SLBT and HSIs remains unclear. Therefore, to validate the usefulness of evaluation methods related to HSIs, a longitudinal study is necessary.

## Conclusions

The MS-SLBT, particularly when using 60 and 40 cm platforms, requires faster buttock-raising speeds, greater heel-bearing force, and higher levels of hamstring muscle activity than the SLBT. Additionally, the 40 cm platform demands the greatest heel-bearing force and is also the most practical for clinical settings, making it highly versatile. These findings suggest that the MS-SLBT has potential as a valuable tool for assessing hamstring functions in high-speed, bi-articular movements.
